# Uniform Distribution and Densification of Jets in Needleless Electrospinning Using Annular Tip Nozzle

**DOI:** 10.3390/polym11081301

**Published:** 2019-08-02

**Authors:** Hongbo Chen, Chuansheng Wang, Imdad Ali, Haoyi Li, Xiaoqing Chen, Weimin Yang, Wenwen Han, Haichao Liu, Dongmei Jiao, Fengfu Yin

**Affiliations:** 1 College of Electromechanical Engineering, Qingdao University of Science and Technology, Qingdao 266061, China; 2 College of Mechanical and Electrical Engineering, Beijing University of Chemical Technology, Beijing 100029, China

**Keywords:** melt electrospinning, needleless, self-organization, linear free surface

## Abstract

Numerous jets can be generated simultaneously on a nozzle by needleless melt electrospinning technology which has the advantages of solvent-free residues and environmental friendliness; and potential industrial application prospects. In this paper, the linear annular tip nozzle was taken as the research object, and the high-speed image acquisition of the jets generation and distribution process of annular tip nozzle was carried out and compared with that of straight-line tip nozzle. The results showed that the repulsive force between the jets caused a slight adjustment in the position of the jets on the free surface, the force between the jets on the annular closed curve canceled each other and eventually reached the equilibrium state, making the position of the jets stable and the distance between the jets the same, and the distance between the jets was related to the intensity of the induced electric field at the tip of the nozzle. Relevant conclusions can provide scientific and practical guidance for the design of needleless electrospinning nozzles on free surface in order to achieve uniform and efficient preparation of ultrafine fibers.

## 1. Introduction

Electrospinning, as a special preparation method of fibers, can prepare polymer fibers with diameters in the range of from nanometers to the submicron scale [1,2,3]. Many functional materials, for application in biochemical protection, tissue engineering, hyperfiltration, wound dressing, catalysis, and biomedicine, have been fabricated by this method [4,5,6,7,8,9,10]. Compared with solution electrospinning, melt electrospinning showed great advantages, e.g., solvent-free residue, high production efficiency, environmentally friendly [11,12,13].

Previously, same as most solution electrospinning, the equipment of melt electrospinning were based on capillary nozzles, which can excite jets at low voltage, but with low efficiency [14]. Later, needleless melt electrospinning was proposed and numerous jets could be generated simultaneously. The needleless melt electrospinning with the slit head was carried out, in which the melt was distributed along a straight line at the slit and then multiple polymeric jets were produced under high voltage electrostatic [15]. The distance between the jets was large, about 5.2 mm, and uneven. A linear laser was used to heat the polymer sheet into melt, making the jets distribute in a linear array under the action of the electrostatic field [16]. The minimum jet spacing was 4.5 mm and there was still unevenness. The list of jet spacing of polymer needleless melt electrospinning is shown in Table 1. In addition, a rotating disc as needleless electrospinning nozzle was used to generate multiple jets by dipping melt, but the jet uniformity was not mentioned [17]. In needleless electrospinning, reducing jet spacing is helpful to increase the output of fiber, and the uniformity of jet spacing is helpful to improve the uniformity of fiber film distribution [18,19]. Therefore, the key point of needleless electrospinning nozzle design is how to make the jet spacing small and uniform. In solution electrospinning, the one-dimensional electro-hydrodynamic theory was established to predict the critical electric field intensity, and the mechanism of “fastest forming instability” was considered to be the cause of the self-organization of the system [20]. However, this theory is not suitable for melt electrospinning and cannot explain the uniform distribution of jets in needleless melt electrospinning under closed linear free surface.

**Figure 1:**
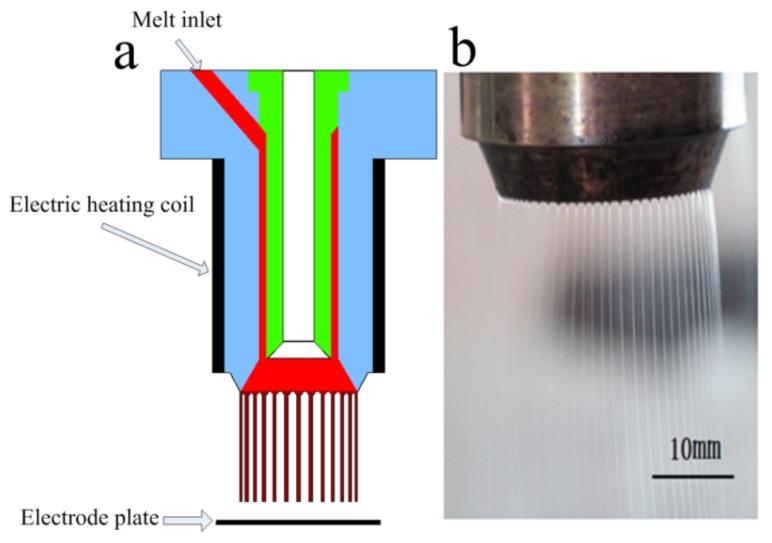
Needleless melt electrospinning of annular tip nozzle: (**a**) schematic, (**b**) photo.

In the previous research, a special needleless melt electrospinning device was proposed by our group. As shown in Figure 1, polymer melt passed through the annular channel inside the nozzle and distributed evenly at the annular closed linear tip of the nozzle, and then multiple jets were formed under the action of high voltage electrostatic field. After self-organization process, the jet spacing became uniform and small, the number of jets could reach dozens, and the spacing could reach 1.1 mm [21]. The jets cooled and solidified to form fibers during the falling process. The multiple jets generated onfree surface of the annular nozzle tip were stable and uniform, which made the fiber diameter distribution uniform. In some applications of fibers, the uniformity of fibers had a great influence on their properties. For example, in drug-sustained-release membranes, uniform fibers helped to improve the drug-sustained-release ability [22,23]. In addition, there was a high demand for uniformity of fibers in the applications of filtration membranes, catalytic supports, photonic and electronic devices, as well as biomedical scaffolds [24]. Therefore, the uniform ultrafine fibers produced by this method will have great potential for applications. Furthermore, this high yield method offered the possibility of industrial production of ultrafine fibers. However, the formation cause of such small and uniform jet spacing was still unclear. In order to reveal the self-organization distribution mechanism of jets in needleless melt electrospinning using annular tip nozzle, a high-speed image acquisition experiment was carried out to study the formation and distribution process of jets under the annular nozzle, and the induced electric field intensity at the nozzle tip was simulated. The different distributions of the induced electric field intensity at the tip of straight-line nozzle and annular nozzle were compared and analyzed. The tip of annular nozzle was closed, while the tip of linear nozzle was open, which led to the differences of self-organization of jets and distribution of induced electric field intensity. Unlike annular nozzle, the open ends of linear nozzles could not effectively restrict the jets, and the jets could not reach a stable equilibrium state. At the same time, the induced electric field intensity of both ends was stronger than that of the middle part, which resulted in the uneven distribution of the induced electric field intensity on the linear nozzle, the uneven distribution of the jets and large jet spacing. The relevant analysis and conclusions could provide scientific guidance for industrialized equipment design of melt needleless electrospinning.

## 2. Experimental

**Figure 2:**
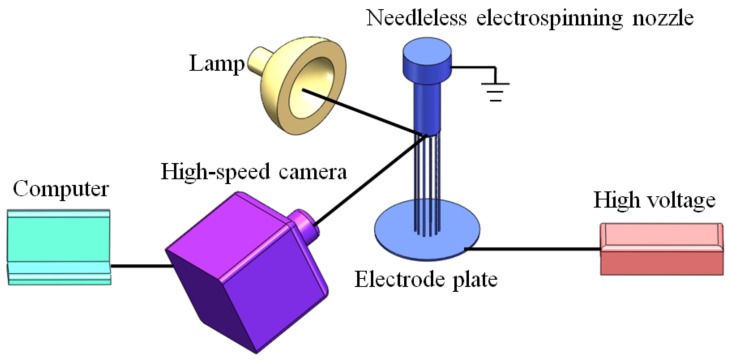
Schematic diagram of high-speed image acquisition device for needleless melt electrospinning process.

The schematic diagram of the experimental apparatus is shown in Figure 2. In this experiment, a nozzle with annular closed tip above-mentioned with diameter of 26 mm was used. Polymer particles were melted by a self-designed micro extruder. The melt was then metered and extruded into the nozzle at the melt flow rate of 12.5 g/h, and evenly distributed at the annular tip after passing through the internal flow path of the nozzle. Polypropylene (PP) (Shanghai Expert in the Developing of New Material Co., Ltd., Shanghai, China, MFI = 2000 g/10 min) was used during the experiment. The high electrostatic voltage, provided by a high voltage electrostatic generator (Tianjin Dongwen Company, Tianjin, China, 0–80 kV) was applied to a disc electrode below the nozzle, while the nozzle was grounded. A high-speed camera (Southern Vision Systems, Inc. (SVSi), Madison, WI, USA, Giga View) was tilted upward and pointed at the annular tip of the nozzle, and the high brightness light source was provided by a lamp.

Firstly, the high-speed camera was connected to the computer and debugged to ensure it worked properly. The high brightness lamp was turned on and shined at the tip of annular nozzle. Then, the high-speed camera was placed to make the shooting direction and the direction of the light remain 90 degrees in the same plane. The shooting angle of the high-speed camera was adjusted and tilted upward about 20 degrees, so that the jet distribution of the entire annular nozzle tip could be captured by the camera. The focal length of the high-speed camera was adjusted to capture the image the annular tip of the nozzle clearly. Next, the high-voltage electrostatic generator was turned on, the voltage was adjusted to the value required by the experiments and the voltage adjustment knob was kept in place, so as to ensure that the high-voltage electrostatic generator could directly reach the required voltage value when it was turned on the next time, and then the high-voltage electrostatic generator was turned off for further use. Finally, the micro extruder was turned on making the melt evenly distribute at the tip of the nozzle. When the melt flowed out steadily, the high-speed camera recording function was enabled and the high-voltage electrostatic generator was turned on with the voltage set by the previous step directly applied to the electrode plate. At this time, jets formed at the tip of annular nozzle. The high-speed image acquisition was performed at a rate of 200 fps. After the jets stabilized, the high-voltage electrostatic generator and computer recording were turned off, and the time needed from the jets generation to the stability was obtained by playing back one frame by one on the computer. The jets distribution under different voltage could be obtained by adjusting the voltage value and repeating the above process. The nozzle temperature was set to 250 °C, spinning distance (distance between nozzle and electrode plate) was set to 100 mm, and applied voltage was set to 20, 25, 30, 35 and 40 kV, respectively.

## 3. Results and Discussion

### 3.1. Theoretical and Experimental Analysis of the Formation of Stable and Uniform Jets

**Figure 3:**
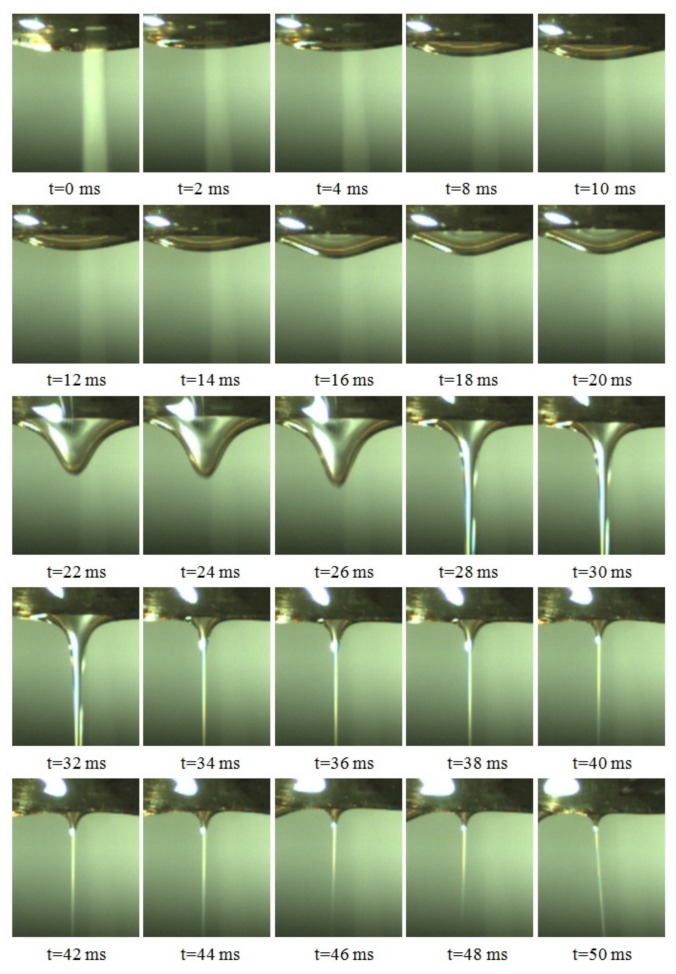
Photos of single jet formation process.

After applying high voltage electric field, the melt at the tip of annular nozzle formed a single jet and gradually evolved into several uniform and stable jets. The formation of a single jet on the free surface of annular nozzle tip is analyzed in this paper. When the applied voltage is 30 kV, in the process of jets increasing, one of the typical jets was selected for analysis. The formation process is shown in Figure 3.

In the high voltage electrostatic field, the surface of the melt as a dielectric was polarized, forming polarized charges that charged the melt surface. The charged droplet in the electric field would be deformed, and the tip charge of the deformed droplet would be concentrated. When the charges accumulated to a certain stage, the sum of the electric field force and gravity on the droplet was greater than the sum of its surface tension and viscous force, a jet would be formed. As can be seen from the Figure 3, the development of the jet underwent a process from large volume to small volume. At the beginning stage, there was a large amount of melt at the tip of nozzle, and viscous force caused the melt around the root of the jet to gather toward its center, leading to the large jet volume, as shown in 0 to 32 ms of Figure 3. As the jet fell and a large amount of melt was removed, the volume of the jet decreased rapidly. When the speed of the melt transported by the extruder was the same as that of the melt carried away by the jet falling, the size of the jet gradually tended to be stable and unchanged, as shown in 34 to 50 ms of Figure 3.

**Figure 4:**
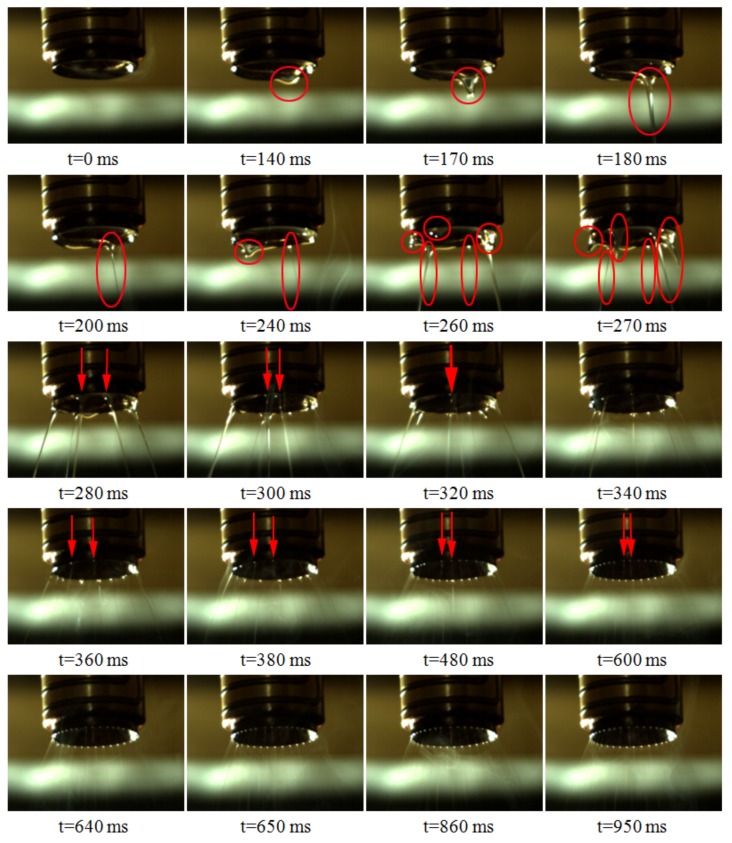
Photos of jets development process when the spinning voltage is 30 kV.

The relevant analysis was extended from the generation of a single jet to the distribution process of the multiple jets at the tip of the annular nozzle. When the applied voltage was 30 kV, the jets lasted for about 950 ms from generation to stabilization, and some of the jet distribution photos are shown in Figure 4. The jet distribution process can be roughly divided into three stages: random jet generation, jets gap filling and uniform distribution.

In the initial stage, the polymer melt was continuously delivered to the internal runner of nozzle by the micro extruder and a uniform melt thin layer was formed on the conical inner surface of nozzle bottom. Due to melt adhesion effect, a large number of melt gathered at the annular tip of nozzle, forming a thick melt ring, as shown in 0 ms of Figure 4. When the high voltage electrostatic generator was turned on, the voltage gradually rose from 0 to 30 kV. With the increase of voltage, the polarization charges on the melt ring gradually increased, resulting in the deformation of the melt ring to produce a single bulge, as shown in 140 ms of Figure 4. As the induced charges accumulated, the bulge developed into a jet. The formation of the first jet in the initial stage took about 240 ms, while the formation of a single jet selected in Figure 3 took about 50 ms. It can be seen that the time required for the formation of these two jets was different. The voltage increased gradually with time, so in the early stage, the electric field intensity was weak, the polarization charges of melt ring were less, electric field force was weak, which caused the jets to fall slowly. When the voltage increased, the induced electric field intensity increased rapidly, the polarization charge density increased, the electric field force strengthened, which caused the jets to fall faster. Part of the induced charges was taken away by the fall of the jets. With the increase of voltage, the induced electric field intensity at the tip of nozzle increased gradually, the induced charges on the melt ring surface increased, and the melt ring deformed at random positions, forming more bulges. Then these bulges formed jets. The jets formed randomly on the free surface of the annular tip, so this stage was called the random jet stage, which ranged from 0 to 270 ms.

In the initial stage of jet number increased, there was a large amount of melt at the tip of nozzle making the volume of jets large, and the high viscosity of melt resulted in adhesion and fusion between jets. After the random jet stage, a large number of melt at the tip of nozzle was taken away, and then the jet volume became smaller. Jets were formed under the same induced electric field, so the surface of jets charged with the same polarity. Like charges repel each other, unlike charges attract, so the jets repel each other. The adhesive force of jet decreased with the decreasing of its volume. In addition, the jets formed on the linear free surface, and were not fixed like the jets formed on needles. The jets were restricted on the annular tip and could be suspended at any position. Therefore, the repulsion between jets enabled the position of jets to change. With the further increase of polarization charge density, the number of jets also increased further, new jets were split between formed jets, and this stage was called jets gap filling stage, approximately ranging from 280 to 480 ms.

**Figure 5:**
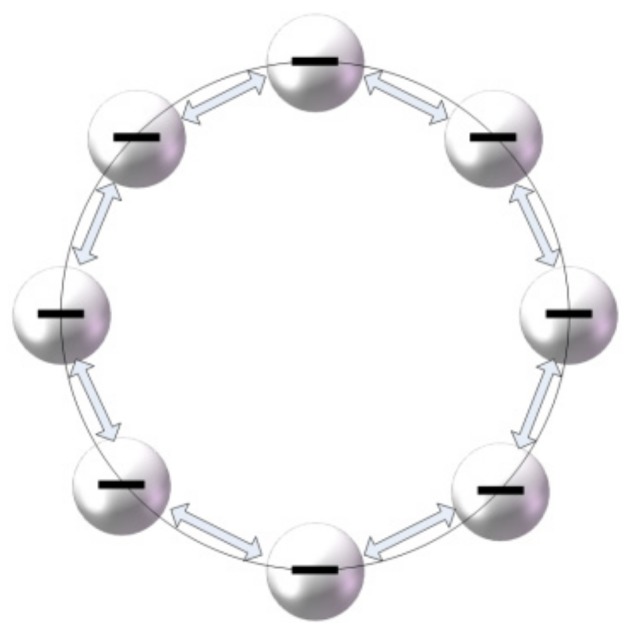
Diagram of force acting on charged jets at annular tip.

**Figure 6:**
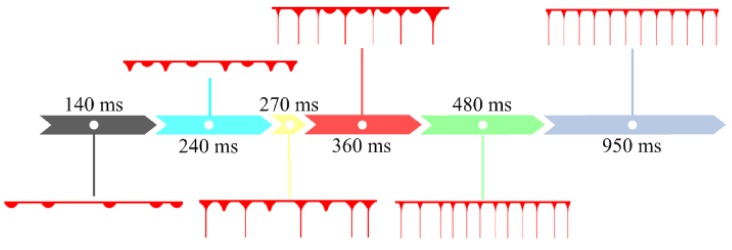
Schematic diagram of jets development process. (For ease of expression, the closed curve is straightened to a straight line).

A part of polarization charges were taken away by a single jet when it fell down. When the size of the jet was constant, the polarization charges taken away were stable. The increase of voltage caused the melt surface to form more polarization charges, which would stimulate more jets, and the jets fell to take away the newly generated polarization charges. When the set voltage was reached, the amount of polarization charges taken away by the continuous falling of the jets was the same as the amount of polarization charges formed by the continuous electrostatic induction, the system would form a dynamic balance, and the amount of jets would no longer increase. At this time, the number of jets reached the maximum. Then the development process entered the third stage, uniform distribution stage, from 480 to 860 ms. At this stage, the position of the jets was further fine-tuned under the action of repulsive force. After constantly adjusting, the size of the jets and the amount of polarization charges carried by each jet were basically the same. Each jet was subjected to two adjacent repulsion forces with the same size. Finally, on the annular closed line, the repulsive forces between jets canceled each other to reach an equilibrium, and the position of jets was fixed, that was, the distance between jets was uniform, as shown in Figure 5. The entire development process of jets is shown in Figure 6. These three stages were not isolated, but continuous, such as the phenomena of adhesion, fusion and movement of jets existed in the whole development stage of jets.

**Figure 7:**
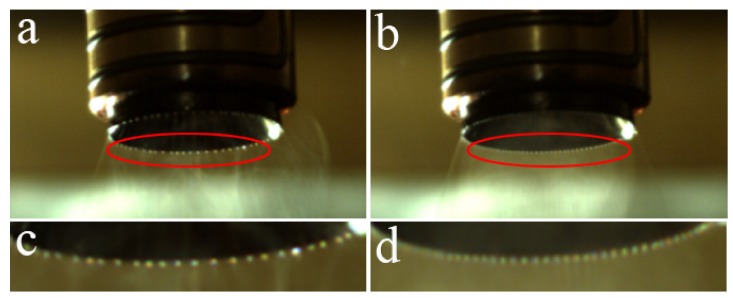
Overall and partial enlarged photosof jet distribution after self-organization when the spinning voltage is 35 kV (**a**,**c**) and40 kV (**b**,**d**).

The effect of applied voltage on jets development was also investigated. The jet distribution after self-organization at applied voltages of 35 and 40 kV is shown in Figure 7. When the applied voltage was 35 kV, it took 600 ms for jets from generation to stabilization, 350 ms less than that at 30 kV. When the applied voltage was 40 kV, the time is further reduced to 450 ms. It can be seen that, the increase of applied voltage accelerates the process of jets self-organization, which makes the jets rapidly reach the state of uniform distribution equilibrium. According to Coulomb’s Law, the interaction force between two static point charges in a vacuum is proportional to the product of the charge, and inversely proportional to the square of the distance between them:

(1) $$F=K\frac{Q_{1}Q_{2}}{r^{2}},$$

where *Q*_1_ and *Q*_2_ are charged quantities of two objects respectively, *r* is the distance between two objects (centers) and *K* is a constant.

When the voltage increased, the induced electric field intensity increased, and the polarized charges on the jets increased accordingly. The repulsive force between jets increased, which promoted the moving speed of jets to be faster. Therefore, the self-organizing distribution process of jets was accelerated, and the dynamic equilibrium state was reached quickly.

It also can be seen from Figure 7 that the number of jets increases with the increase of voltage. This is because the polarizing charge carried by a single jet is finite, in order to balance the increased induced charge brought by the increased of voltage, more jets are produced by the equal amount of melt. Therefore, it can be inferred that, on the premise that the air between the nozzle and the electrode plate is not broken down, more jets will be generated through taking measures to increase the induced electric field intensity of nozzle tip.

**Figure 8:**
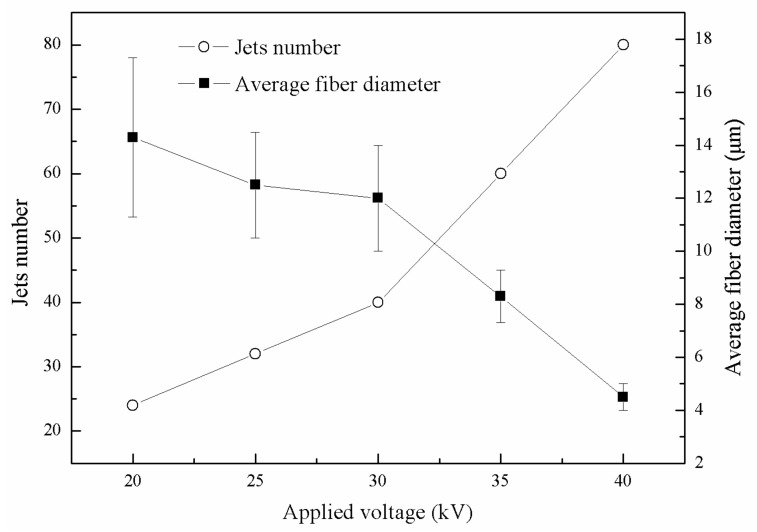
Jets number and average fiber diameter at different applied voltages.

**Figure 9:**
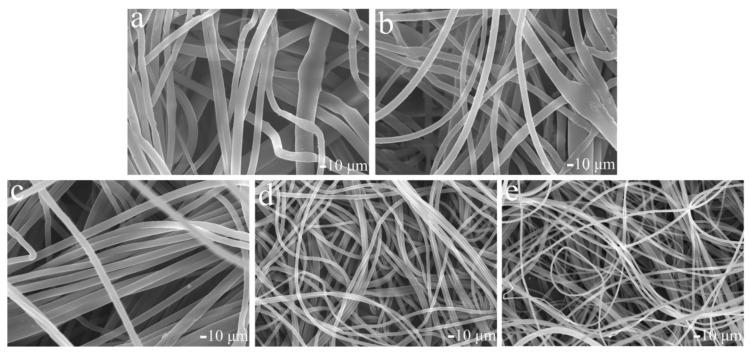
SEM images of melt electrospun fibers at different voltages: (**a**) 20 kV, (**b**) 25 kV, (**c**) 30kV, (**d**) 35 kV, and (**e**) 40kV.

The melt supply was constant, so the increase of the number of jets would reduce the volume of each jet and made the fiber thinner. In addition, when the voltage increased, the electric field force on the jets increased with the increase of polarized charge density of the jets, and then the jets speed and melt tensile rate increased, further thinning the fibers [25]. The number of jets increased with the increase of voltage, reaching 80 at 40 kV. Meanwhile, the average diameter of fibers decreased with the increase of voltage, as shown in Figure 8. The morphologies of the fibers were investigated by scanning electron microscopy (SEM), as shown in Figure 9. The standard deviation of fiber diameter distribution also decreased with the increase of voltage, which was because the increase of voltage enhanced the repulsion between jets, accelerated the self-organization process of jets, and promoted the uniform distribution of melt flow on each jet, resulting in uniform distribution of fiber diameter. Therefore, high induced electric field intensity could help improve the spinning efficiency and further refine the fibers.

**Figure 10:**
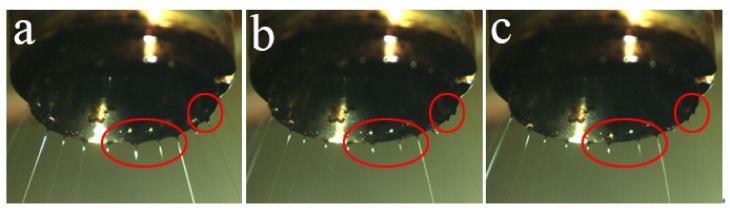
Photos of local jet distribution at different times when the spinning voltage is 20 kV: (**a**) 550 ms, (**b**) 650 ms, (**c**) 750 ms.

Based on the above conclusions, when the applied voltage was relatively low, the induced electric field intensity of nozzle tip was weak, resulting in a decrease in the number of jets and an increase in jet spacing. The local jet distribution of the nozzle tip at 20 kV is shown in Figure 10. It can be seen from the figure that the number of jets does not change after 550 ms, and remains only about 24. However, the jet spacing is still not uniform until 750ms, and the jets continue to be in the position adjustment state. This is because the large distance between jets weakens the repulsion force between jets, and the small number of jets leads to the large volume of a single jet. As a result, it becomes difficult for repulsion force to overcome the viscous force between melt and nozzle to promote the jet movement. Eventually, self-organization slows down.

**Figure 11:**
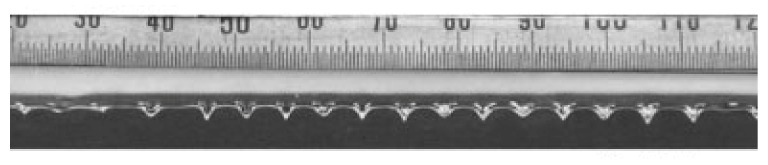
Melt needleless electrospinning at straight-line open free surface [16]. Reprinted with permission.

The needleless melt electrospinning experiment on a non-closed curve such as a straight line also indirectly proves the correctness of the above conclusion, as shown in Figure 11. It can be seen that the jets’ spacing on the linear free surface is large and unevenly distributed. This is because the jets at both ends of the straight line are only subjected to the repulsive force of the adjacent jets on one side, and there is no jet on the other side. As a result, the jets’ spacing at both ends of the line is larger than that in the middle part.

**Figure 12:**
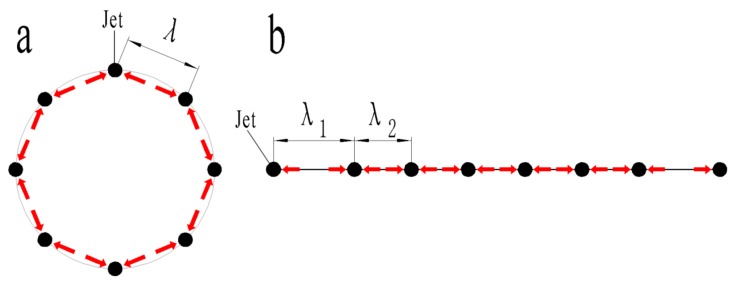
Diagram of interaction forces between jets: (**a**) annular closed curve; (**b**) straight-line open curve.

The schematic diagram of the relevant principle is shown in Figure 12, black dots stand for jets, red arrows stands for repulsion, λ stands for jets spacing. On the annular closed curve, the force of each jet is the same, making λ the same, but on the open straight-line, the jets at both ends are only subjected to one-way repulsive force, which increases the jet spacing, making λ_1_ > λ_2_. A similar phenomenon was observed in solution electrospinning. For example, in array capillary electrospinning, although the needle position was fixed, the jets repelled each other, and the jets close to the outside bent outward under the action of repulsion [26]. The same phenomenon had also been observed in the free surface needle-free solution electrostatic spinning [20].

**Figure 13:**
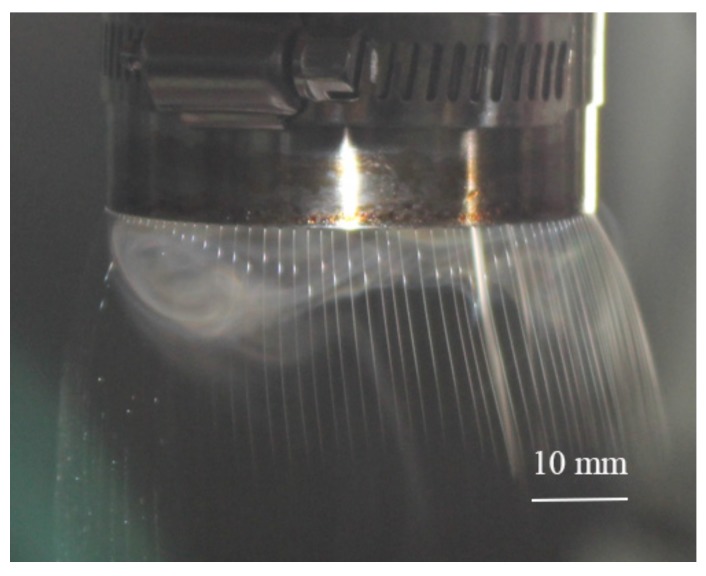
Melt needleless electrospinning with 52 mm annular tip nozzle.

Experiments on annular closed curve tip nozzle with enlarged diameter further confirmed the correctness of the above conclusions. As shown in Figure 13, when the annular tip diameter of the nozzle was expanded to 52 mm, the melt could still form uniformly distributed and stable multiple jets under the high voltage electric field due to the equilibrium of interaction forces between jets.

### 3.2. Theoretical Analysis of the Formation of Jets with Small Spacing

From the above comparison experiments, it also can be seen that besides the uniform and stable jets spacing, the jets spacing on the annular tip is smaller than that on the straight-line tip. Previous studies had shown that the intensity of the induced electric field at the nozzle tip was negatively correlated with the jet spacing in melt needleless electrospinning [21]. Therefore, the induced electric field intensity of annular and straight-line tip was studied through the finite element method (FEM) simulation to analyze the formation causes of jets with small spacing. In this paper, the relevant simulations were performed based on the FEM software Comsol Multiphysic.

**Figure 14:**
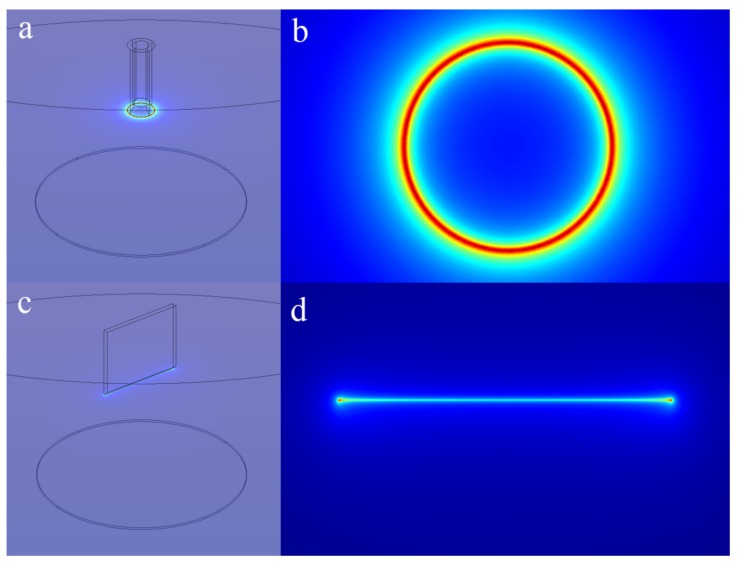
Distribution of the induced electric field: (**a**,**b**) annular tip; (**c**,**d**) straight-line tip.

The experimental device models of the annular tip nozzle and the linear tip nozzle were established, respectively. The spinning voltage was set to 30 kV and the other parameters were the same as the above melt electrospinning experiment for simulation calculation. For the annular-type tip nozzle, the maximum induction field intensity was generated at the tip of the nozzle, and distributed evenly in the whole circumference, as shown in Figure 14a,b. The maximal induction field intensity is represented by the red color. However, for the straight-line-type tip nozzle, the distribution of induction field intensity at the tip of the nozzle was not uniform, and the maximum induction field intensity was concentrated at both ends, as shown in Figure 14d. For straight line type nozzle, compared to the middle smooth part, the charge density at both ends was higher and the induced electric field nearby was stronger, so that the tip discharge easily occurred. Therefore, on the premise of no breakdown, the voltage applied on the electrode plate with the annular nozzle could be higher than that with the straight line type nozzle, so as to form stronger induction electric field intensity at the tip of circular annular nozzle and thus form smaller jets spacing. The concentration and uniform distribution of induced charge at the tip of the annular nozzle provided favorable conditions for the formation of jets with small spacing and uniform distribution. It can be inferred that the strong and uniform distribution of the induced electric field at the nozzle tip is the prerequisite for the formation jets with small spacing and uniform distribution in needleless melt electrospinning.

## 4. Conclusions

A high-speed camera was used to collect images of the formation and distribution of the jets at the tip of an annular nozzle. And the correlation simulations based on the finite element software COMSOL aiming at analyzing the induced electric field intensity under nozzles with different types of tips were carried out. The research in this paper can provide scientific and useful guidance for the design of needleless electrospinning nozzle on free surface. The relevant conclusions are as follows:

1. The experimental results showed that the development of the jets in needleless melt electrospinning with annular nozzle could be divided into three stages: random jet generation, jets gap filling and uniform distribution;

2. The induced electric field intensity at the tip of the nozzle determined the number of jets to be formed, and the repulsive force between the jets caused a slight adjustment in the position of the jets on the free surface. The annular shape of the tip was closed curve, making the repulsive force of the adjacent jets counteract each other, and reach the equilibrium state. Finally, the position of the jets was in a stable state. Macroscopically, the distance between the jets was the same, and the position did not change. With the increase of applied voltage, the polarized charges increased, resulting in more jets and faster stable state. On the contrary, when the number of jets was small and the distance between jets was large, and the repulsive force was weakened, which resulted in the jet continually in the state of position adjustment and instability;

3. The needleless melt electrospinning based on nozzle with annular tip could generate jets with a larger density than that with the straight-line tip nozzle. The simulation results showed that the annular tip could form a strong and uniform induced electric field, but the induced electric field caused by the straight-line tip was not uniform.

In a word, for needleless melt electrospinning based on nozzle with linear tip, the precondition of forming stable and dense multi-jets is that the tip curve should be smooth and closed, so that the electric field is uniformly distributed, and the repulsive force counteracts each other to reach equilibrium.

## Figures and Tables

**Table 1:** List of jet spacing of polymer needleless melt electrospinning.

Polymer	Equipment Type	Jet Spacing [mm]	Ref.
Polypropylene (PP)	Rod	6.3	[15]
Polypropylene (PP)	Cleft	5.2	[15]
Poly(ethylene-co-vinyl alcohol)(EVOH)	Polymer sheet melt by line laser	4.5	[16]
Polypropylene (PP)	Annular tip nozzle	1.1	[21]
